# Problems in Cross-Cultural Use of the Hospital Anxiety and Depression Scale: “No Butterflies in the Desert”

**DOI:** 10.1371/journal.pone.0070975

**Published:** 2013-08-09

**Authors:** Gemma A. Maters, Robbert Sanderman, Aimee Y. Kim, James C. Coyne

**Affiliations:** 1 Health Psychology Section, Department of Health Sciences, University of Groningen, University Medical Center Groningen, Groningen, The Netherlands; 2 Interdisciplinary Studies in Human Development, Graduate School of Education, University of Pennsylvania, Philadelphia, Pennsylvania, United States of America; 3 Institute for Health, Health Care Policy and Aging Research, Rutgers, the State University of New Jersey, New Brunswick, New Jersey, United States of America; Catholic University of Sacred Heart of Rome, Italy

## Abstract

**Objective:**

The Hospital Anxiety and Depression Scale (HADS) is widely used to screen for anxiety and depression. A large literature is citable in support of its validity, but difficulties are increasingly being identified, such as inexplicably discrepant optimal cutpoints and inconsistent factor-structures. This article examines whether these problems could be due to the construction of the HADS that poses difficulties for translation and cross-cultural use.

**Methods:**

Authors’ awareness of difficulties translating the HADS were identified by examining 20% of studies using the HADS, obtained by a systematic literature search in Pubmed and PsycINFO in May 2012. Reports of use of translations and validation studies were recorded for papers from non-English speaking countries. Narrative and systematic reviews were examined for how authors dealt with different translations.

**Results:**

Of 417 papers from non-English speaking countries, only 45% indicated whether a translation was used. Studies validating translations were cited in 54%. Seventeen reviews, incorporating data from diverse translated versions, were examined. Only seven mentioned issues of language and culture, and none indicated insurmountable problems in integrating results from different translations.

**Conclusion:**

Initial decisions concerning item content and response options likely leave the HADS difficult to translate, but we failed to find an acknowledgment of problems in articles involving its translation and cross-cultural use. Investigators’ lack of awareness of these issues can lead to anomalous results and difficulties in interpretation and integration of these results. Reviews tend to overlook these issues and most reviews indiscriminately integrate results from studies performed in different countries. Cross-culturally valid, but literally translated versions of the HADS may not be attainable, and specific cutpoints may not be valid across cultures and language. Claims about rates of anxiety and depression based on integrating cross-cultural data or using the same cutpoint across languages and culture should be subject to critical scrutiny.

## Introduction

The Hospital Anxiety and Depression Scale [1] is one of the most widely used questionnaires in clinical and health psychology worldwide, outside of the United States where it has not won as much favor. It has been translated into 78 languages [2] for use in both western and non-western countries. The HADS is the most frequently used measure of mood disturbance in cancer care, where it has been applied in two-stage screening, assessment of severity of mood disturbance, and for validation of other measures [3]. It was originally designed for clinicians with the aim of providing a short screening instrument assessing psychopathology in non-psychiatric medical patients. Based on the assumption that scores on existing mood scales were confounded with somatic complaints in medically ill patients, the developers of the HADS excluded items seen as overlapping with symptoms of a somatic disorder [1]. Explicit reference to psychiatric symptoms was avoided and colloquial British English was chosen for some items, notably “I get a sort of frightened feeling like ‘butterflies’ in the stomach”, and response options varied across items in terms of both wording and keying. The Depression subscale (7 items) was based mainly on symptoms of anhedonia, rather than depressed mood, because the authors assumed that the former symptoms would respond better to antidepressants. The Present State Examination [4], together with research into clinical manifestations of anxiety neurosis [5], provided the basis for the 7-item Anxiety subscale. Reviews of the psychometric properties of the HADS have generally concluded that it has adequate sensitivity, case finding ability, concurrent validity and internal consistency [6], [7].

The HADS continues to enjoy international use and wide endorsement as one of the best available measures of depression and anxiety both for screening purposes and assessment of symptom severity, but several difficulties are now being identified in the same literature. It is our purpose to explore the implications of these difficulties for translation and cross-cultural use and to evaluate whether investigators have handled the HADS with appropriate sensitivity to issues. The goal is to evaluate whether non-equivalence of the HADS across languages and cultures might explain problems in the generalizability of cutpoints and consistency of factor-structures that have been reported.

### Issues Raised in the Recent Literature Concerning the HADS

Vodermaier et al’s [8] review noted a troublingly broad, inconsistent range of optimal cutoffs obtained across studies, ranging from 8–22 for total score and 5–11 for depression and anxiety subscales. Singer et al [9] also noted varying cutpoints between studies for the depression subscale, and suggested re-calculation of different cutpoints for distinct groups of patients. Carey et al [10] reported a wide range of recommended thresholds in their recent review of validation studies performed in cancer patients. A Danish study [11] unexpectedly found lower mean HADS scores in a sample of breast cancer patients relative to women of the general population, a result that challenges either the presumed greater levels of depression among cancer patients than in the general population, or the validity of the HADS as a means of establishing relative levels of depression.

Cosco et al’s recent review [12] of 50 studies concluded that factor-structures of the HADS varied across studies and within populations, with the particular factor solutions ranging from one to four factors, with findings dependent upon the specific analytic strategy employed. Inconsistencies were greatest with cancer patients, the medical population in which the HADS is the most widely used measure of anxiety and depression. Cosco et al concluded that the original intention of the HADS having a two factor-structure distinguishing between anxiety and depression had not been achieved, and that the HADS should be interpreted as an assessment of emotional distress that does not distinguish between anxiety and depression. Cosco et al recommended that “the absence of psychometric robustness suggests that researchers should interpret subscale scores with caution or use the total score.”

In a pair of commentaries Coyne and Van Sonderen [13], [14] accepted Cosco et al’s conclusions concerning the basic factor-structure of the HADS, but disputed a recommendation for continued use of the HADS as a screening instrument, noting the inconsistencies in the cutpoints that were obtained within and across populations. They proposed that some problems might stem from decisions made in construction of the HADS, and particularly its deliberately varying response keys. They noted the consistently anomalous factor loading of item 7 (‘I can sit at ease and feel relaxed’), pointing out that it is a positively valence item, but with a reversed response key and different anchors than the item that just proceeded it. Coyne and Van Sonderen [14] expressed doubt that even an exceedingly alert patient would notice and be responsive to these changes in what was being asked. To answer consistent with the intention of the design of the HADS, patients would have to be attentive to sudden changes back and forth between positive and reverse worded items and in the available response options:

…*six items alternate between positive and reverse worded items indicating negative affect, but the seventh item breaks with this pattern. Furthermore, going from item to item, the first available response option shifts from “most the time,” to “definitely as much,” to “very definitely and quite badly” to “as much as I always could,” to “a great deal of the time,” to “not at all,” to “definitely.” The “not at all” is for the item “I feel cheerful” and the “definitely” is for the item “I can sit at ease and feel relaxed.” A number of items are ambiguous as to whether they refer to actual level of negative affect or to a comparison with ‘usual’.*

We would add that when it comes to translating the HADS, it might prove difficult to preserve the comparability of positive versus reverse worded items, as well as the equivalence of the varying response key options across languages. Paralleling the problems of patients completing the HADS, translators might simply overlook these transitions, fail to capture them adequately in a second language, or they might improvise in an effort to compensate for problems that were recognized.

### Four Different Dutch Versions of the HADS

Our concerns about translation and cross-cultural use of the HADS were prompted when we discovered four different Dutch versions of the HADS [15]–[18]. The four Dutch versions have different content for five (items 5, 7, 9, 11 and 13) of the 14 items, different response options in nine items (items 1, 2, 3, 4, 5, 7, 10, 11 and 14), different ranges of scores (0–4 or 1–3) and different timeframes (one week versus four weeks). Yet, we could find no indication in the published studies depending on a Dutch translation of the HADS that these multiple versions existed or which version was used, either among primary research studies using one of the versions, or in secondary discussions or integrations of results of the primary studies. The finding of four different Dutch versions was worrisome because the distinctions between these versions could conceivably prove substantial and there is little reason to presume that results could be generalized from one version to another. For generalizability across these four translations to hold, it would have to be assumed that results were not substantially influenced by differences in content, response options, or time frames. It would be extraordinary if this were the case. Thus, recommendations for cutpoints for Dutch translations are highly unlikely to generalize across versions, and integration of data from Dutch versions with the original English version or translations into other languages is likely problematic, particularly if the goal is identification of a cross-culturally valid cutpoint. We sought to determine how the translation of the HADS is being handled in other languages, whether potential problems were noted, and how they were being addressed.

### Challenges in Translating the HADS

A review of translation methods by MAPI Research Trust [19] concluded that recommendations for cross-cultural translations of questionnaires need further development and that a multistep approach was needed to obtain high quality translations. A checklist was provided in the review to assess the methods used in a translation process and to list actions taken. Producing a dependable, high quality translation is costly and labor intensive [19], [20]. Several other papers already have paid attention to the complexity of producing a high quality translation, ways to reach equivalence across different languages and cultures, and problems that might arise in the translation process [21]–[24]. Adequate *cultural adaptations* of instruments are not easily achieved and with questionnaires usually not designed with anticipation of the issues posed for translation, it is difficult to ensure that items in a translated instrument are conceptually equivalent to the original version [25]. If not addressed carefully, the influence of language or culture might manifest in each of several ways. One possibility is a shift in mean scores. Another possibility is diminished validity, because the translated item measures something else than intended in the original version [26], as represented in different validity correlates. Subtle differences between questionnaires caused by translation of items or response options could lead to incomparable cutpoints.

MAPI Research Trust in France is responsible for the distribution of HADS translations. They state “the author has selected MAPI Institute as exclusive linguistic validation company to ensure the production of harmonized and consistent language versions” [2]. Yet, MAPI did not carry out all translations and validations. The original developers of the HADS intended to make the items easy to translate into other languages [27]. But the question is whether they succeeded, and whether the apparent benefits of the reliance on colloquial British English for construction of items remain when the instrument is translated into other languages. An earlier guideline by Brislin et al [28] cautioned against use of colloquialisms in a questionnaire because of the risk of subsequent difficulties in achieving an equivalent translation. So, reliance of the developers of the HADS on colloquial British language complicates the translation process, in addition to the existing complexity of achieving an adequate translation in itself.

In preliminary work, we had sent an email inquiring about translation procedures to a sample of investigators. As anticipated, several of the colloquial items turned out to be difficult to translate into some languages. For instance, considerable effort was put into translating the item “I get a sort of frightened feeling like ‘butterflies’ in the stomach” into Omani Arabic dialect. The investigators recognized that they had to capture the intended feeling, and chose to do this in the audiotaped delivery of the item. In addition, the author of an Arabic version explained to us: “A lot of difficulties because this question of the butterflies appeared not only strange but rather funny to many Arabic-speaking individuals”. Translation of the response options turned out difficult in some languages as well. This is what the author of a Punjabi version replied: “The response options were difficult to translate, to get appropriate gradations between ‘all of the time’ and ‘most of the time’. The same word was commonly used in Punjabi for both of these responses”. Although not systematic, this preliminary work encouraged us to look further into the awareness of these issues on the part of investigators who were using translated versions and in reviews that integrated results from translated versions with results from the original English version. In sum, we had obtained preliminary indications that the HADS is not as easy to translate as intended by the developers and that unacknowledged problems might exist in translated versions.

### Cultural Awareness of Investigators in their Usage of the HADS

We next looked for remarks in HADS literature concerning problems that might have been caused by using translated versions. Surprisingly few concerns were expressed in literature about the use of translated versions of the HADS. Herrmann [6] noted that scores on translated versions of the HADS might be influenced by cultural factors. As one of the possible explanations for the diverging thresholds, Carey et al [10] referred to the translated versions of the HADS used cross-culturally in the studies they reviewed. They noted that only one of the ten studies validating the HADS for use with cancer patients had used the original English-language version and that different translations might yield different factor-structures and optimal cutpoints. A study by Martin et al [29] in patients with coronary heart disease in three different countries suggested a three-factor structure. But the factor-structure turned out to be different among the three countries. Wang et al [30] identified issues in factor-structure in the Chinese version of the HADS as possibly caused by difficulties in the translation of the HADS into Chinese. Similarly, a study by Chan et al [31] indicated a two-factor structure, but also the loading of item 7 (‘I can sit at ease and feel relaxed’) on depression. Citations indicate the same Chinese translation was used in both studies. El-Rufaie and Absood [32] concluded that differences in cutpoints of the Arabic HADS relative to the English version might have been caused by linguistic or cultural factors. Research conducted in Oman [33] compared HADS scores with the results of the Composite International Diagnostic Interview, in patients with Traumatic Brain Injury and found a sensitivity of 53.8% and specificity of 75.9%, but with an optimal cutpoint of only four. It was concluded that the poor performance of the HADS might have arisen in the process of translating the questionnaire into the Omani dialect. Chaturvedi [34] pointed out how the results of studies with translated HADS versions in Asian participants could have been affected by cultural differences, commenting on a paper by Nayani [35]. In their recent review, Cosco and colleagues [12] acknowledge the possibility of translation issues causing heterogeneity in factor-structures. But the tables and source papers in their article suggest different HADS language versions were nonetheless integrated.

Overall, few concerns were expressed about the use of translated HADS versions and subsequent consequences, which made us suspicious of the awareness of investigators of these problems. We were concerned whether investigators who used a translation of the HADS identified the source of a translated version they used and measures taken to ensure proper validation. Van Widenfelt et al [21] observed that quite often articles fail to report the origin of translated questionnaires. In addition, we examined if authors of reviews integrate data from diverse cultures and translations and acknowledge difficulties in doing so. While HADS is our specific focus, other instruments, particularly those constructed in colloquial language, might pose the same issues when translated and used cross culturally.

## Methods and Results

### Reports of HADS Translations and Validity Studies in Papers Originating from Non-English Speaking Countries

We were encouraged to examine how explicit and accurate investigators reported in their article about the translated version of the HADS, its provenience or, if it was translated by the investigators themselves, how validity was assured. A comprehensive search was performed in the Pubmed and PsycINFO databases in May 2012. Keywords were (“HADS”) OR (“HAD scale”) OR (“hospital anxiety” AND “depression” AND (“scale” OR “scales” OR “score” OR “scores” OR “subscale” OR “subscales” OR “sub-scale” OR “sub-scales”). After removal of duplicates, and citations for book chapters and comments and letters to the editor, 4555 references were left. To reduce the scope of the task, every fifth (20%) of the remaining abstracts were examined by one of the authors (GAM), and a research assistant. They examined 913 abstracts and removed references of papers that were written in another language than English (79) or in which the HADS had not actually been used, but only cited (4). For the remaining 830 abstracts, the country in which the research was conducted was recorded. For 15 papers the country could not be determined, because it was not mentioned in the abstract and the full text was not available either on the web or through interlibrary loan. A total of 345 articles originated from an English speaking country, of these 69% (237 papers) originated from the UK. Other identified English speaking countries were the USA, Australia, Canada and New Zealand.

A total of 470 papers originated from non-English speaking countries (58%). Available full texts of the papers were examined (417). Country, indications of the use of translated HADS versions and documentation on the source of a translated version and its validity were recorded. Table 1 shows the results of our examination. The first column indicates the country in which the study was executed. Per country the total number of papers examined are reported (second column), as are the reports of authors on the language version used (third column). The number of citations of studies validating a particular language version and the number of citations of the 1983 study by Zigmond and Snaith [1] are indicated in the last two columns. As a specific illustration using results presented in Table 1, 34 papers originating from Norway were examined in total. Yet, only 9 out of the 34 papers indicated a Norwegian version of the HADS was used. The other 25 papers reported nothing about the version used. In the Norwegian case 30 out of 34 papers cited Zigmond and Snaith, but only 14 out of 34 papers cited a validation study of a Norwegian version of the HADS.

**Table 1:** Reports of translated HADS versions used, citations of the Zigmond and Snaith 1983 study and citations of validation studies with non-English HADS versions, by investigators in non-English speaking countries.

Source country of papers	Number of papers examined per country	Reporting of use of translated versions of the HADS	Languages of HADS translations as reported by investigators	Number of citations of Zigmond and Snaith (1983)	Citations of validation studies with non-English versions
Austria	4	1	German	4	1
Belgium	4	0	**	4	0
Brazil	12	4	Portuguese	12	6
Canada (French speaking part)	2	2	French - Canadian	2	2
China	19	18	Chinese, Chinese – Cantonese, Mandarin	16	17
Denmark	9	2	Danish	8	2
France	29	4	French	21	11
Germany	38	22	German	30	29
Greece	11	3	Greek	11	5
Holland	55	25	Dutch	45	33
Iceland	5	4	Icelandic	5	4
India	5	2	Malayalam, Urdu	5	3
Iran	6	6	Iranian, Persian	5	6
Israel	4	1	Hebrew	3	1
Italy	24	10	Italian	23	9
Japan	25	20	Japan	23	19
Jordan	2	0	**	2	1
Kosovo	1	0	**	1	0
Lithuania	4	0	**	4	2
Malaysia	3	2	Malay, Bahasa Malay, Mandarin- Chinese and Tamil	2	1
Morocco	1	1	Arabic	1	1
Nigeria	1	0	**	1	1
Norway	34	9	Norwegian	30	14
Palestine	1	0	**	1	0
Poland	3	0	**	2	0
Portugal	6	5	Portuguese	6	6
Russia	1	0	**	0	0
Singapore	2	0	**	2	1
South Korea	8	6	Korean	7	6
Spain	18	11	Spanish	16	11
Sweden	43	11	Swedish	42	9
Switzerland	11	6	German	9	7
Taiwan	7	2	Chinese-Cantonese	4	3
Thailand	2	1	Thai	1	1
Turkey	17	11	Turkish	13	14
**Total**	**417**	**189**		**361**	**226**

** The article(s) did not report the language version of the HADS used.

On the whole, explicit reports of the use of a translated version of the HADS were outnumbered by articles making no statements at all about the version used; in only 45% did investigators state that they used a translated version of the HADS in their study and indicated the language. Of all papers from non-English speaking countries 46% did not cite a validation study in the language of their country and yet 13% did not even cite Zigmond and Snaith [1]. In conclusion, although the HADS was frequently used in non-English speaking countries, less than half of the papers originating from non-English speaking countries reported which particular HADS version was used and slightly more than half of the papers did report validation in the language to which the HADS was translated.

### Integration of Data from Different Language Versions of the HADS in Reviews

Seventeen reviews, including two meta-analyses [3], [6]–[8], [12], [36]–[47], that integrated studies with at least two different language versions of the HADS, were next extracted from our database. These papers were examined by GAM and AYK for the strategies that the authors reported to deal with different language versions, the way different versions were compared and reports of possible problems and corrective actions concerning language or culture. Table 2 summarizes the results of our examination. The Table shows which language versions were compared to each other, and in what way (column 4 and 5). In the last column of the Table all comments by the authors of the reviews, if any, about language or culture are listed.

**Table 2:** Reports of translated HADS versions used, and of corrective actions and qualifications concerning language and culture in reviews that integrated studies with different language versions of the HADS.

Reference	Type of paper and paper objective			Number of studies in paper involving the HADS	Reports of type of comparison of different HADS versions		Reports of languages of HADS, if studies from non-English countries were integrated.		Reports of corrective actions or qualifications concerning language and culture
[7]	Literature review; to review the validity of the HADS			71	Evaluation of factor analyses, subscale correlations and internal consistency and the calculation of a mean cut point for HADS-D and HADS-A separately, with a range of sensitivity and specificity, in cancer patients.		Arabic, Chinese (Cantonese), Dutch, English, French, French Canadian, German, Japanese, Italian, Norwegian Swedish, Portuguese, Spanish, Swedish.		“The variation in both optimal cut-off values and sensitivity and specificity might be due to differences in diagnostic systems, ‘gold standard’ instruments, HADS translations used …….”. “It has been recommends that Cronbach’s coefficient alpha should be at least.60 for a self-report instrument to be reliable [55]. This demand was fulfilled in all studies of HADS in various translations that report data on internal consistency. Similar findings of internal consistency from different translations of HADS supported the robustness of the instrument”.
[41]	Meta-analysis; to assess the HADS’ ability to detect anxiety and depressive disorders			25	Pooled specificity and sensitivity estimates and summary receiver operating characteristic curves for different cut points and for three disorders separately (major depressive disorder (MDD), generalized anxiety disorder (GAD) and any depressive disorder (ADD)).		MDD: eight English, one Flemish or French (performed in Belgium), one German and one Japanese. GAD: four English, one Italian, one Spanish and one not specified (but study was performed in Nigeria). ADD: one Chinese, one Dutch, eleven English, one German, one Italian, one not specified (executed in Nigeria).		“When we explored underlying causes of heterogeneity in the case of cut point ≥8, we found that the diagnostic odds ratio did not vary according to the ……, whether a translated version of the HADS was used (P = .72), and…….”
[36]	Literature review: to assess instruments for measuring psychological consequences of false-positive screening mammography			5	Different HADS-versions and their psychometric properties were not compared. The authors state about the five studies reviewed “These do not report pre-testing, test-retest reliability, or analyses of internal consistency in these settings”.		Not stated in the paper. Original sources found one Dutch, two English, one Norwegian, and one Swedish.		“….The language of a questionnaire must be kept up to date as the linguistic value of words and terms can take on new meanings over time. Both the wording of the items and the construct behind the measures could be different if the measures had been developed more recently”.
**Reference**	**Type of paper and paper objective**			**Number of studies in paper involving the HADS**	**Reports of type of comparison of different HADS versions**		**Reports of languages of HADS, if used in non-English countries.**		**Reports of corrective actions or qualifications concerning language and culture**
[6]	Literature review: to assess the acceptability to patients, reliability and validity of the HADS.			Not stated in the paper.	Studies on the HADS were reviewed by the author.		The author states “Empirical data are available from twenty-five countries outside the United Kingdom”. Some languages are noted specifically in the text when relevant (e.g. English, German, Urdu).		The author makes several remarks on language and culture, for instance: “The scale can be considered sufficiently validated for use in Arab countries, China, France and Belgium, and Germany and Switzerland. For several other countries, only partial validity information is available. ………. This indicates that, despite probable identity of psychometric properties, HADS scores may be different in countries with different cultural patterns of perceiving and expressing emotions, which is an important issue when transferring the scale to new cultural settings”.
[37]	Systematic literature review: to assess the prevalence of depression in cancer patients and hospice populations in literature.			15	None.		Not stated in the article. Original sources indicate one Chinese, thirteen English and one Italian.		None.
[3]	Meta-analysis: to inspect diagnostic validity and the practical appropriateness of the HADS in cancer patients.			24	Diagnostic validity (pooled sensitivity and specificity) for anxiety, depression and any psychiatric disorder were examined.		Not stated in article. Review of the original sources lead to estimate twelve English, two French, four German, three Italian, two Japanese, one Turkish, and one either Afrikaans or English (since this study was carried out in South Africa).		None.
[38]	Systematic literature review: primarily to find the prevalence of depression in patients with myocardial infarction but also to compare prevalence between different measures.			5	The authors did not perform a psychometric comparison of different HADS versions. They compared prevalence of depression between different measures.		Not stated in the article nor original sources. However, four English and one Swedish probable.		None.
**Reference**	**Type of paper and paper objective**			**Number of studies in paper involving the HADS**	**Reports of type of comparison of different HADS versions**		**Reports of languages of HADS, if used in non-English countries.**		**Reports of corrective actions or qualifications concerning language and culture**
[40]	Systematic literature review: to assess the psychometric properties of screening tools for symptoms of depression in AMI survivors.			5	Evaluation of the reliability and validity of several instruments, based on predefined criteria.		Not stated in the article nor original sources. However, two Dutch and three English probable.		None.
[8]	Systematic literature review: to examine the Psychometric properties of screening measures for affective, anxiety, and adjustment disorders in cancer patients			41	The psychometric properties were evaluated and rated with decision rules (regarding reliability, criterion measure and validity).		In the paper is stated that twenty were non-English: three French, three German, one Greek, one Hungarian, one Indian, two Italian, five Japanese, one Persian, one Slovenian, one Swedish and one Turkish). Also, one was from a South European population. The rest (twenty) are probably in English.		“The most extensive validation existed for the HADS, and this was the case across disease types and stages as well as across languages and cultures. The scale has been extensively tested against criterion standards.”
[39]	Systematic literature review: to find out what measures have been used for depression cases in studies involving palliative cancer patients.			76	Frequency of usage for depression assessment.		Not stated in the paper. However, from the list of references we could understand that translations in several different languages were used, e.g. Greek, Japanese and Italian. But also the English version.		None, but regional differences in the usage of HADS are mentioned.
**Reference**	**Type of paper and paper objective**			**Number of studies in paper involving the HADS**	**Reports of type of comparison of different HADS versions**		**Reports of languages of HADS, if used in non-English countries.**		**Reports of corrective actions or qualifications concerning language and culture**
[42]	Literature review; to assess studies in which the HADS is being validated against the SCID and to compare recommended cut points with actual use of cut points by investigators.			10	Two authors reviewed all studies, according to predetermined criteria (e.g. on sample appropriateness, reporting of precision estimates and reliability of the SCID).		The country (setting) of the source papers is reported in a Table. From this table we could understand that translations in German, Italian, Japanese, Turkish, Flemish or French (study performed in Belgium), were used. But also the English version.		“Of the 10 cancer validation studies identified for the HADS using the SCID as a gold standard, only one was conducted using the English language version of the HADS. Validation studies of different language versions of the HADS have been associated with different factor structures [56] and optimal thresholds for identifying caseness [7]. It has been suggested that HADS thresholds may vary cross culturally as a result of variations in the symptomatic presentation of anxiety and depression [6], [57], [58]. For example, it has been suggested that culture may influence whether depression is expressed in emotional and psychological terms or whether it is manifested as physical symptoms [59]. As different items within the HADS focus either on physical or psychological symptoms, endorsement of different combinations of items in a given population will alter the specificity and sensitivity of a given threshold for defining caseness [60]. Therefore, one might expect that the threshold for defining caseness may vary between cultures depending on the way in which cultural norms influence respondents’ answers”. ….“Validation studies conducted with HADS in cancer populations offer little consistency with respect to the thresholds; this is possibly caused by variability within the patient-with-cancer populations in terms of culture, disease stage, treatment status and type of disease across the studies”
**Reference**		**Type of paper and paper objective**	**Number of studies in paper involving the HADS**		**Reports of type of comparison of different HADS versions**	**Reports of languages of HADS, if used in non-English countries.**		**Reports of corrective actions or qualifications concerning language and culture**	
[12]		Systematic literature review: to review the factor structure of the HADS.	50		Two authors reviewed all studies and reported on sample, as well as methods and results of factor analyses. Results were presented by methods used (EFA, CFA, IRT) and population.	Not stated in the article. Original sources indicate Chinese, Dutch, Spanish, Norwegian, Portuguese, French, Uzbek, German, Greek, Hungarian, Swedish and Japanese versions were included, as well as the original English version.		None.	
[43]		Systematic literature review: to assess evidence on anxiety level and contributing factors of women undergoing treatment for breast cancer.	3, but only two of them were com-pared to each other.		Two independent reviewers examined all papers and extracted data using two standardized data extraction tools (JBI-MAStARI).	A Swedish and original English version of the HADS were reported.		None.	
[44]		Narrative literature review: to assess the suitability of the HADS as a screening tool in an alcohol-dependent population.	28 to examine factor analysis, 5 to examine test-retest reliability and 26 to determine internal consistency reliability.		Factor analysis and reliability of the HADS in several populations were assessed by two authors.	Factor analysis; Dutch, German, French, Spanish, Uzbek, Chinese, Greek, Norwegian, Portuguese and English. Test-retest reliability: English, Uzbek, Chinese, Greek, Portuguese. Internal consistency reliability; English, Uzbek, Spanish, Greek, Hungarian, Iranian, Norwegian, Portuguese, Chinese.		“The Pais-Ribeiro et al. (2007) study [61] looked at a multiclinical Portuguese sample and the low test-retest score may be due in part to the translation of the HADS or some other criterion”.	
**Reference**		**Type of paper and paper objective**	**Number of studies in paper involving the HADS**		**Reports of type of comparison of different HADS versions**	**Reports of languages of HADS, if used in non-English countries.**		**Reports of corrective actions or qualifications concerning language and culture**	
[45]		Systematic literature review; to review questionnaires in perinatal populations.	3		A combined checklist was used to assess study quality (e.g. reporting on sample and reliability).	Original English version, Uzbek and Nigerian.		“Despite excellent sensitivity and specificity, low internal consistency and discrepancies in factor structure and the prevalence of probable anxiety disorder identified using the recommended cut-off of 8 are a concern, although these may be due to cultural or methodological differences”.	
[46]		Narrative literature review; to review studies on depression in primary cerebral glioma.	10		Four categories of clinical associations were assessed; patient-related, tumor-related, treatment-related and outcome-related.	A Spanish version and the original English version (9 studies).		None.	
[47]		Systematic literature review; to review the validity of distress measures, in cancer care.	13		Psychometric properties of self-report measures, in studies comparing the questionnaires with a SCID, were reviewed in different phases of the cancer trajectory; pre-treatment, during active treatment, post-treatment and during palliative care.	Pre-treatment: Original English, Japanese and Italian. Active treatment; Original English and Italian. Post-treatment: Japanese, Italian and German. During palliative care: Japanese and Original English.		None.	

Seven papers did not mention that they included studies with several different translated versions [3], [12], [36]–[40], although we could determine that they did so by examining citations and source articles. Few concerns or problems were reported about reliance on translations. Bjelland et al [7] raised concerns on the reliability of the HADS across translations. But they argued against this being a problem, citing Cronbach’s coefficient alphas of ≥.60 in all studies. However, such reliability does not establish comparability. Bjelland et al also calculated a mean cutpoint >8 on the anxiety and depression subscales for cancer patients. Yet, examining the original source papers we discovered that the mean cutpoint was calculated from one study with an Italian version, two studies with a French version, one study with a Japanese version and five studies with the original British version (including one study executed in South Africa). In the original source paper of the Italian study no specific information is provided on the origin or quality of the used translation [48]. One of the French studies reported that the HADS was translated into French by Zigmond and Snaith [1] and validated by Lepine [49] and Razavi [50], but it is difficult to evaluate the quality of the translations from the information that was provided. The Japanese study mentioned a back translated Japanese version by Kitamura [51]. Thus, examining the original source papers did not yield a clear picture of the quality of the different translated versions, and so the calculated mean cutpoint across countries could be of dubious value. Herrmann [6] warned that scores could vary across cultures, and validity studies have not been performed for all translated versions. So, the review by Herrmann [6] stands out as an exception in which it is stated that culture or language has to be taken into account. Vodermaier et al [8] concluded there is considerable evidence for HADS validity in different languages, relative to other measures used for research in cancer care, although cutpoints differed between studies. A recent meta-analysis by Brennan et al [41] inspected the possible contribution of translation to heterogeneity, with a fixed cutpoint. Based on a diagnostic odds ratio of.72 they decided against it. On the other hand, the paper by Carey et al [42] explicitly mentioned how culture might influence HADS thresholds. And Meades and Ayers [45] referred to cultural or psychometric factors contributing to problems with the internal consistency, factor-structures and cutpoints.

In sum, attention paid to translation and cross-cultural issues is limited in the reviews that we examined. The authors of most review papers indiscriminately compared results obtained with different language versions of the HADS without acknowledgment.

## Discussion

Our discovery of four different Dutch versions of the HADS triggered concerns over whether cross-cultural and translation issues cause problems in the wide usage and interpretation of this instrument worldwide. Our concerns were consistent with problems increasingly raised in HADS literature concerning varying cutpoints and factor-structures. The aim of this paper was to investigate the possibility that cross-cultural and translation issues are underlying to the reported problems in HADS literature.

Examination of a sample of abstracts from papers on studies using the HADS showed this questionnaire was used more often in non-English speaking countries than in English speaking countries. Thus, integrative reviews and meta-analyses of cutpoints and correlates of the HADS that do not distinguish between studies conducted in different languages are relying more on translated versions than the original English version. Yet, most papers originating from non-English speaking countries did not report the version of the HADS used, and only slightly more than half of all papers report whether it was validated in the language of the participants. In the reviews and meta-analyses we examined, cross-cultural issues were addressed in only seven of the seventeen papers [6]–[8], [41], [42], [44], [45]. Others uncritically combined studies conducted in different cultures and languages [3], [12], [36]–[40], [43], [46], [47]. Thus, cultural awareness of investigators concerning the HADS turned out unsatisfactory in our sample.

We believe that the inattention to problems in translating the HADS can explain at least some of the problems in varying cutpoints across studies as well as inconsistencies in factor-structure. These problems can be compounded when data from translated versions are integrated across studies in narrative and systematic reviews. However, documentation exists of varying cutpoints and factor-structures in when studies are limited to English-speaking populations with the unaltered original instrument, and so use of the translated HADS alone cannot explain more pervasive problems.

This paper indicates considerable room for improvement in terms of transparency and accuracy on the part of investigators regarding the origin of version of the HADS used. This is likely a more general issue in the reporting of studies using translated questionnaires [21]. We strongly recommend that journals publicize requirements for explicit reporting of the information concerning translation and revalidation in any cross-cultural use of the HADS or other translated questionnaires. According to the Scientific Advisory Committee of the Medical Outcomes Trust [52] for others to be able to review the quality of the translation and cultural adaptation of a questionnaire, the following information should be made available by the developers: how linguistic and conceptual equivalence were reached, whether any differences exist between the original and the new version, and how inconsistencies where dealt with. Acquadro et al [19] further provide a checklist to assess the information reported in articles concerning the process of translation and revalidation by. To be able to use this checklist, detailed information on the method of translation used, the translators involved and the qualification, any communications with the developer(s) of the original version, pilot testing and “International Harmonization” is needed. Analogously, we suggest that investigators dependent on an already translated tool to report in their papers at the minimum: the language or dialect into which the HADS was translated, how the translated version was obtained, whether the quality of the translation process and the result of this process were reviewed and if a validation study was conducted with the translated version. Lacking information on the quality of a translation and validation of a questionnaire, readers cannot be certain that problems in the language or the cross-cultural usage of the HADS did not bias or even invalidate the results of the study. Yet, published studies reviewing or using the HADS have consistently assumed that different versions are comparable enough so that any differences can be ignored.

We caution that our review was not exhaustive, but was based on a sampling of 20% of papers with results dependent on the HADS. However, our efforts meet the Black Swan criterion: we think that we have found sufficient documentation of problems in the translation and interpretation of the HADS to reject the null White Swan hypothesis of no problems in the translation or cross-cultural interpretation of the HADS. Yet, we need to start to ensure that our measures – as the HADS - across languages/cultures are measuring exactly the same so that we can trust comparisons of data collected in different languages.

The problems that we have identified with the cross-cultural use of the HADS may not be specific to this instrument, but endemic to translated versions of other instruments, and particularly in those deliberately constructed in colloquial language, these problems may even be more pervasive. The Edinburgh Postpartum Depression Scale [53] embraces British colloquial language with the item ‘Things are getting on top of me’, which must strike many Americans as odd and confusing. Similarly, the item on the Beck Depression Inventory [54], ‘I feel sad and blue’ will perplex respondents confronting the item in languages like Italian where “blue” does not have the affective connotation as in English. Translators would seem to do best to avoid attempting literal translations of colloquialisms, but then run the risk of not being able to establish exact equivalency at the item level, and possibly the scale level. Based on the limited number of reports we obtained for investigators using the HADS cross-culturally, we suspect that considerable improvisation occurs and therefore inconsistency in results in the translation of other scales.

In conclusion, we think the issues currently being raised in HADS literature concerning inexplicably varying factor-structures and cutpoints might very well be created in part or amplified by translation and cross-cultural problems. Results obtained with translated versions of the HADS should be treated with caution. Because most investigators in this study were not explicit on the way the translated version was acquired and how validation was ensured, there is no guarantee that authors handled the HADS in a proper culturally sensitive way. Our results strongly suggest that readers of published cross-cultural studies should have some skepticism about the validity of findings and that future publications should better document exactly what was done to ensure the cross-cultural validity of translated versions and generalizations from results obtained in other cultures and languages. If other questionnaires are being handled in the same way by investigators, this warning applies to these measures too.
